# Sleep disorders in higher education students: Modifiable and non-modifiable risk factors

**DOI:** 10.14744/nci.2021.44520

**Published:** 2022-07-01

**Authors:** Beatriz Minghelli

**Affiliations:** Escola Superior de Saúde Jean Piaget do Algarve - Instituto Politécnico Jean Piaget do Sul – Instituto Piaget; Research in Education and Community Intervention (RECI), Silves, Portugal

**Keywords:** Anxiety, depression, internet addiction, sleep quality, university students

## Abstract

**OBJECTIVE:**

Poor sleep quality among higher education students is a world public health problem that can lead to a decrease of the concentration and consequently the academic performance. This study aimed to determine sleep quality among higher education students and to verify its association with internet addiction and psychological disorders (anxiety and depression).

**METHODS:**

The sample comprised 148 higher education students of the south of Portugal, being 108 (73%) female, aged between 18 and 54 years old. The measuring instrument included a sociodemographic questionnaire, the Pittsburgh Sleep Quality Index, the Internet Addiction Test, and the Hospital Anxiety and Depression Scale.

**RESULTS:**

Fifty-five (37.2%) students had a good sleep quality, 77 (52%) poor sleep quality, and 16 (10.8%) a severe sleep disorder. Forty-one (27.7%) students said that they went to bed between 12:00 am and 12.30 am and 37 (25%) between 11 pm and 11.30 pm. The sleep duration was 7:08±1:31. Fifty (48.1%) students who classified with poor sleep quality classified themselves as good sleepers (p≤0.001). Internet addiction was not associated with poor sleep quality. Students who present anxiety and/or depression symptoms had 0.31 (CI: 0.135–0.71; p=0.005) more probabilities to have sleep disorders compared to those who did not show these symptoms.

**CONCLUSION:**

This study found that most of the analyzed students present a poor quality of sleep and this was associated with a presence of anxiety and/or depression. It becomes necessary to develop sleep hygiene education programs for prevention and treatment of sleep disturbances in this population.

## Highlight key points

- The vast majority of students presented some type of sleep disorder.
- Although most students had sleep disturbance, a high percentage of them classified their sleep quality as good.
- Anxiety and/or depression symptoms increase the probability of students to present sleep disorders.

Sleep is a necessary condition for good mental health, a necessary factor to obtain better academic performance, as it can contribute positively to student learning [1–5]. The recommended sleep duration for adults must be at least 7 h/night [6]. Insufficient sleep decreases general alertness, impairs attention, and slows cognitive processing [3, 7–9].

Poor sleep quality among university students is a public health problem all around the world. University students are considered a vulnerable risk group to poor sleep quality because of several factors that are involved in the academic life and social challenges. Some of these factors include the fact that many students move to other cities, and as such, are free for the first time from all parental control, and can choose their own bedtime [5, 9, 10], the increased amount of time spent on studying or extracurricular activities and consequently increased academic stress [9, 11–15], the increase of environmental noise because of shared living spaces [9, 11, 13], and others factors.

In recent decades, the insertion of new technologies has transformed cultural habits and the lifestyle of university students worldwide. University students are recognized as one of the groups with greater sleep deprivation and one of the most technologically-oriented [16]. Some evidence suggests that poor sleep quality has been associated with internet addiction [17]. The use of internet devices during the night period and the brightness of the light that they project on the retina are some factors that can cause changes in sleep patterns [18, 19].

Besides that, the sleep quality is most commonly affected by stress and anxiety [12, 20], which are common among university students as well as the poor sleep can increase the risk for mental illness [3, 9, 21].

Since it is a current and highly relevant topic for public health, and because studies at a national level are unknown, the objective of this study was to determine the sleep quality among higher education students living in Portugal and to verify its association with internet addiction and psychological disorders (anxiety and depression).

## MATERIALS AND METHODS

This study had an observational and cross-sectional nature to obtain data about sleep quality among higher education students living in the south of Portugal.

This research was approved by the Research in Education and Community Intervention (RECI), Piaget Institute research center (approved in April 2020) and by School Direction. Written informed consent was obtained from all students who participated in this study.

### Population

The population of this study consisted of 168 higher education students of all sexes and any age, who enrolled in the academic year 2019–2020 in the School of Health Jean Piaget School of Health in Algarve, Piaget Institute of Silves Institute.

The School of Health Jean Piaget School of Health in Algarve is located in the city of Silves, Southern Portugal. There are three health courses in this school: Nursing, physiotherapy, and osteopathy.

The sample size was determined using an estimated mean injury prevalence of 50%, and assuming an error margin of 3% with a 95% confidence interval (CI). Using these assumptions, the minimum sample size contained 146 students [22].

The research inclusion criteria defined students of any course, who freely agreed to participate in the research and thus duly signed the informed consent form.

### Measurement Instrument

The measuring instrument included a sociodemographic questionnaire, the Pittsburgh Sleep Quality Index (PSQI), the Internet Addiction Test (IAT), and the Hospital Anxiety and Depression (HAD) Scale.

### Sociodemographic questionnaire

This questionnaire included questions about gender, age, relationship status, and academic year, if they have children, if they work and if so in what period (full-time or part-time), and physical activity practice.

### PSQI

The PSQI is a validated self-questionnaire that evaluated the sleep quality and disturbances over a period of 1 month [23].

A global score of PSQI more than 5 values shows a diagnostic sensitivity of 89.6% and specificity of 86.5% for the distinction between good and poor sleep quality [23]. Regarding psychometric data in the validation of the instrument to the Portuguese population, Cronbach’s α value for the seven components was 0.70, which reveals a good internal consistency [24].

This instrument presents 19 individual items generate, grouped into seven component scores, and five questions rated by the bedpartner or roommate. These latter five questions are not accounted for in the final score, there is only used for clinical information [23].

The components evaluated subjective sleep quality (C1), sleep latency (C2), sleep duration (C3), habitual sleep efficiency (C4), sleep disturbances (C5), use of sleeping medications (C6), and daytime dysfunction (C7). Each component is scored on a scale between 0 and 3, and then these points of each component are summed to yield a global PSQI score, which has a range of 0–21 points [23, 25].

The total score until 4 points indicates good sleep quality, a score between 5 and 10 points indicates poor sleep quality, and a score of more than 10 indicates a severe sleep disorder [23].

### IAT

IAT is a reliable and Portuguese valid measure of the extent of a person’s involvement with the internet (addictive use of the internet), developed by Young [26], that comprises 20 items that measure mild, moderate, and severe impairment of classification of addictive behavior. Each of these items is rated on a 6-point Likert scale: 0 – does not apply, 1 – rarely, 2 – occasionally, 3 – frequently, 4 – often, and 5 – always. To get the total score, you must sum all the scores for the answers [27].

There are different cutoff points for diagnosing IA with IAT. The first cutoff criteria was proposed by Young in 1998 [28] and the second and more recent (2011) cutoff criteria were proposed by the same author in 2011 [27]. This study used the more recent cutoff criteria that with the interval between 0 and 30 points, the addicted is classified as normal range, 31–49 as mildly addicted, 50–79 as moderately addicted, and 80–100 as severely addicted [27].

### HAD Scale

The HAD was developed by Zigmond and Snaith and measure the intensity of anxiety and depression in non-psychiatric environments and the validated Portuguese version was proposed by Botega et al. [29].

The HAD scale presents good sensitivity, specificity, and internal consistency in assessing anxiety and depression symptoms [30].

The HAD presents a total of 14 items divided into two scales: Seven items measure anxiety (HADS-A) and the others seven measure depression (HADS-D). All questions refer to how the individual has been feeling in the past week. Each item is scored from 0 to 3, depending on the response, and the maximum score is 21 points for each scale. The sum of all the answers gives a final score for each scale and a score until seven values indicate the absence of anxiety or depression, a score between 8 and 10 indicates possible anxiety or depression, and a score equal or more than 11 values indicates the presence of anxiety or depression [29, 30].

### Data Analysis

Statistical analysis was performed with Statistical Package for the Social Sciences (SPSS), version 26.0.

Descriptive statistics were performed in the first approach. Chi-square was used to test for a significant association between categorical variables. Binary logistic regressions, based on the enter methods, and the corresponding CIs were calculated to assed the influence of the included variables on the quality of sleep.

Statistical significance was set at 0.05.

The cuts of the numeric variable “age” took into account the value of the median. The “marital status” variable was grouped into two classes: The people living with another person (married and fact unmarried) and the other living without a partner (single and divorced). The variable “internet addiction” included mildly, moderately, and severe addicted). The anxiety and/or depression presence included possible case and presence of anxiety and/or depression. The “sleep quality” variable was grouped into two classes that include good quality of sleep and poor sleep quality that the last included poor sleep quality and severe sleep disorder.

## RESULTS

The sample comprised 148 higher education students (fulfilling the representativeness of the study population), being 40 (27%) male and 108 (73%) female, aged between 18 and 54-years-old (26.61±7.69). Regarding the course, 91 (61.5%) students were enrolled in the nursing course, 44 (29.7%) physiotherapy, and 13 (8.8%) osteopathy. Considering all courses, 53 (35.8%) students attended the 1^st^ year of the course, 39 (26.4%) were enrolled in the 2^nd^ year, 20 (13.5%) in the 3^rd^, and 36 (24.3%) in the 4^th^ year. Concerning to marital status, 125 (84.5%) students were single, 16 (10.8%) were married, 5 (3.4%) lived together, and 2 (1.4%) were divorced. Thirty-two (21.6%) students had children. Regarding to having a job during the course, 70 (47.3%) students studied and worked. Seventy-two (48.6%) performed some type of physical activity.

Sixty-six (44.6%) students were not classified as internet addicts, 66 (44.6%) were classified as a mildly addicted, 16 (10.8%) as a moderately addicted, and nobody was classified as severely addicted.

Forty-five (30.4%) students present anxiety and/or depression symptoms (included possible case and presence). One-hundred four (70.3%) did not present anxiety, 27 (18.2%) revealed possible case of anxiety, and 11 (11.5%) presence of anxiety. Regarding depression, 136 (91.9%) did not have depression, 8 (5.4%) had depression symptoms, and only 4 (2.7%) students presented a depression.

The data obtained in PSQI indicated that 55 (37.2%) students had a good sleep quality, 77 (52%) a poor sleep quality, and 16 (10.8%) a severe sleep disorder. The score of PSI varied between 0 and 22 points (6.53±3.82).

**Figure 1:**
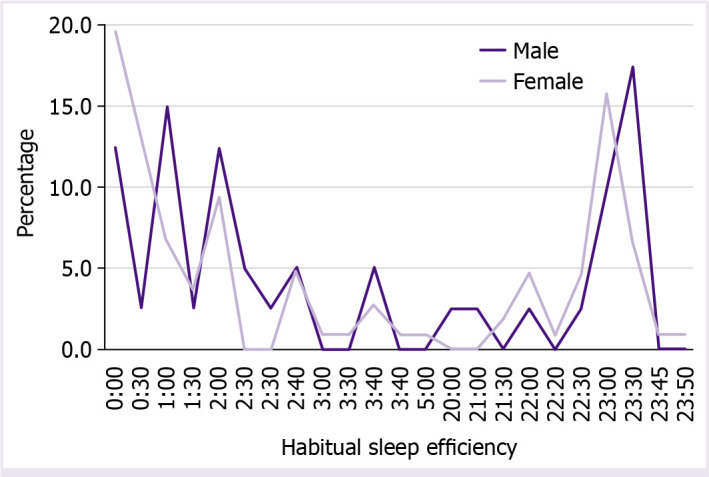
Habitual sleep efficiency by genders.

**Figure 2:**
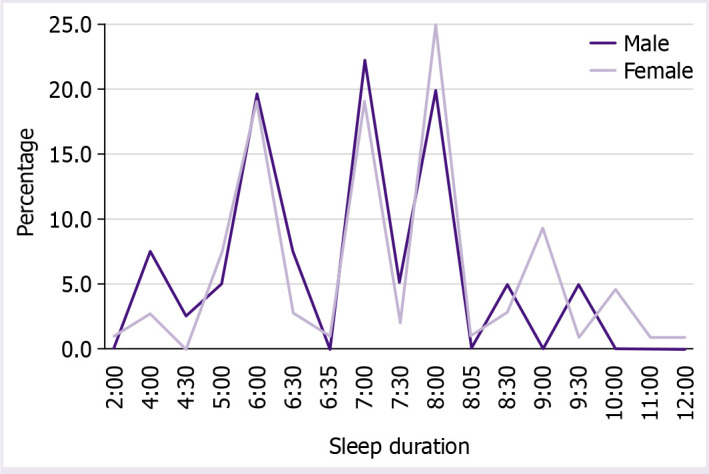
Sleep duration by genders.

Regarding data of habitual sleep efficiency (time to go to the bed), the majority of the individuals of this sample said that they went to bed between 12:00 am and 12.30 am (41; 27.7%), following between 11 pm and 11.30 pm (37; 25%), 1:00 am and 1:30 am (18; 12.2%), 2:00 am and 2:30 am (18; 12.2%), 10:00 pm and 10:30 pm (14; 9.5%), 3:00 am and 3:30 am (9; 6.1%), 4:00 am and 4:30 am, 8:00 pm and 9:30 pm (4; 2.7%), and 5:00 am (1; 0.7%). Figure 1 shows the habitual sleep efficiency by genders and Figure 2 shows the duration of sleep in male and female individuals.

Table 1 shows the frequency and percentage of PSQI answers, referring to the component to which the question corresponds (C1–C7).

**Table 1:** Frequency and percentage of PSQI answers

PSQI questions	PSQI answers			
	Mean±Standard deviation		Minimum–Maximum	
1. When have you usually gone to bed? (C4)	9:13±10:27		20:00–5:00	
2. How long (in minutes) has it taken you to fall asleep each night? (C2)	26.32±25.62		2–200 min	
3. When have you usually gotten up in the morning? (C4)	8:21±1:34		5:00–14:00	
4. How many hours of actual sleep do you get at night? (This may be different than the number of hours you spend in bed) (C3; C4)	7:08±1:31		2–12 h	
5. During the past month, how often have you had trouble sleeping because you…	Not during the past month (%)	Less than once a week (%)	Once or twice a week (%)	Three or more times week (%)
a. Cannot get to sleep within 30 min (C2)	25.7	33.1	20.3	20.9
b. Wake up in the middle of the night or early morning (C5)	27.7	29.1	23	20.3
c. Have to get up to use the bathroom (C5)	31.1	35.1	20.3	13.5
d. Cannot breathe comfortably (C5)	80.4	14.2	2	3.4
e. Cough or snore loudly (C5)	77	10.1	6.1	6.8
f. Feel too cold (C5)	48.6	33.8	13.5	4.1
g. Feel too hot (C5)	46.6	31.1	16.2	6.1
h. Have bad dreams (C5)	36.5	36.5	17.6	9.5
i. Have pain (C5)	60.1	23.6	12.2	4.1
j. Other reason (C5)	–	57.1	42.9	–
7. During the past month, how often have you taken medicine (prescribed or “over the counter”) to help you sleep? (C6)	93.2	2.7	1.4	2.7
8. During the past month, how often have you had trouble staying awake while driving, eating meals, or engaging in social activity? (C7)	73	17.6	6.8	2.7
9. During the past month, how much of a problem has it been for you to keep up enthusiasm to get things done? (C7)	42.6	31.1	20.3	6.1
	Very good (%)	Fairly good (%)	Fairly bad (%)	Very bad (%)
6. During the past month, how would you rate your sleep quality overall? (C1)	16.9	54.1	23.6	5.4
Bed partner	No bed partner or room mate (%)	Partner/roommate in other room (%)	Partner in same room but not same bed (%)	Partner in same bed (%)
10. Do you have a bed partner or roommate?	71	3.4	0.7	25
If you have a roommate or bed partner, ask him/her how often in the past month	Not during the	Less than once	Once or twice	Three or more
you have had:	past month (%)	a week (%)	a week (%)	times week (%)
a. Loud snoring	62.8	20.9	7	9.3
b. Long pauses between breaths while asleep	76.7	16.3	4.7	2.3
c. Legs twitching or jerking while you sleep	37.2	18.6	27.9	16.3
d. Episodes of disorientation or confusion during sleep	76.7	18.6	4.7	–
e. Other restlessness while you sleep	–	–	–	–

C1: Subjective sleep quality; C2: Sleep latency; C3: Sleep duration; C4: Habitual sleep efficiency; C5: Sleep disturbances; C6: Use of sleeping medications; C7: Daytime dysfunction; PSQI: Pittsburgh sleep quality index.

Table 2 shows the association between the classification obtained on PSQI and the classification of the first component of PSQI that is the self-reported sleep quality. These data revealed that 48.1% of students who believe they have a good quality of sleep were classified through the PSQI as poor quality of sleep (p≤0.001).

**Table 2:** Association between the classification of PSQI and the self-reported sleep quality

PSQI classification	Subjective sleep quality (C1)		df	p
	Good quality (include very good+fairly good) (%)	Bad quality (include fairly bad+very bad) (%)		
Good sleep quality	51.9	2.3	1	≤0.001
Poor sleep quality and severe sleep disorder	48.1	97.7		

PSQI: Pittsburgh sleep quality index.

Table 3 shows the relationship, obtained from the application of the binary logistic regression model, between the sleep quality and the analyzed variables in this study.

**Table 3:** Relationship between the event the presence of poor sleep quality and modifiable and non-modifiable risk factors

Variables	Odds ratio_crude_	CI 95%	p
Gender (male*) female	1.81	0.82–4.01	0.142
Age group (≥26-years-old*) until 25-years-old	1.21	0.62–2.37	0.576
Marital status (alone*) with another person	1.32	0.52–3.37	0.561
Worked (yes*) no	1.13	0.58–2.19	0.730
Have children (no*) yes	1.68	0.76–3.71	0.202
Practice of physical activity (no*) yes	1.29	0.67–2.53	0.446
Year of course (past years – 3^rd^ and 4^th^ years*) 1^st^ year – 1^st^ and 2^nd^ years	1.11	0.55–2.20	0.776
Internet addiction (absence*) presence	0.62	0.32–1.21	0.162
Anxiety and/or depression (absence*) presence	0.31	0.135–0.71	0.005

CI: Confidence interval; *: Class reference.

## DISCUSSION

This study found that sleep problems were very frequent in higher education students, noting that 52% of students in the analyzed sample present a poor sleep quality and 10.8% a severe sleep disorder, totaling 62% of sleep disorder. Comparing with the data of others studies using the same measurement instrument and European sample, similar results were obtained on Schlarb et al. [14] study which revealed that 42.8% of 2831 students in Luxembourg and Germany had a poor sleep quality and 17.9% had severe sleep problems, with a total of 60.7%.

Regarding Asia, Najem et al. [31] evaluated 644 Lebanese universities and 56.5% of the students had a poor quality of sleep. Lower values (27.8%) were obtained in Wang et al. [32] study that evaluated 6085 medical students in Mongolia, China. Cheng et al. [20] analyzed a sample of 4,318 university students in Taiwan and 54.7% were classified into the poor sleep quality group. Siddiqui et al. [3] revealed a prevalence of 74.2% of poor sleep quality in 318 medical students, in Saudi Arabia.

In South America, Mesquita and Reimão [33] evaluated 710 Brazilian students and a total of 60.4% of respondents were classified by poor sleepers. Serra-Negra et al. [34] also evaluated Brazilian students (n=183), and 35% of students present poor sleep quality and 42% a severe sleep disorder, totaling 77%, a higher value than the obtained in this study.

In Africa, Seun-Fadipe and Mosaku [12] study data showed a 50.1% of poor sleep quality in 505 Nigerian university students.

All studies referred to above used the PSQI as a measurement instrument, and as we have seen that the quality of sleep in university students is affected in most continents. The sleep quality affects student’s physical and mental health, and consequently their learning capacity. Our sample involved students in the health area, who at various times will do internships and may make mistakes. Thus, this problem may influence the community in the form of accidents and medical error.

Our score of PSI varied between 0 and 22 points (6.53±3.82). Similar data were showed in Mesquita and Reimão [33] study (6.5±2.6; Brazil) and in Siddiqui et al. [3] study (6.79±3.06; Saudi Arabia). Lower values were obtained in Wang et al. [32] (4.46±2.18) and in Wang et al. [13] study (4.91±2.67), both performed in China. Al-Kandari et al. [11] performed your study in Kuwait and the median sleep quality score was 7, higher compared with all these cited studies.

The majority of the students in our sample go to the bed at midnight (habitual sleep efficiency). The reality between countries is different, and in Portugal, most people start their workday at 9 am, thus they can and go to bed later. The mean bedtime on weekdays of Norwegian university students was 23:15 h [35]. Despite being countries with different climates and light during the day, the mean values of students going to bed were very close.

The sleep duration in our students was 7:08±1:31. Equal data were present in a meta-analysis study [10] with a total of 57 studies, with 82,055 university Chinese students included in the meta-analysis (mean of sleep duration was 7.08) and similar data were obtained in Schlarb et al. [14] study in Germany and Luxembourg (mean=7) and in Sivertsen et al. [35] with Norwegian university students (7.24±1.26). Considering sleep duration, the most of the students involved in those previous studies are complying with the recommended minimum hours for slepping [6]. However, Lawson et al. [1] evaluated 153 medical students at the University of Ghana and the mean duration of night sleep was only 5.7±1.2 h.

An interesting fact observed in this study was that almost half (48%) of the students who were classified, by the application of the measurement instrument, as having a poor quality of sleep, stated by self-report that they had a good quality of sleep. The same was observed in Lawson et al. [1] study showed that only 5.9% of students admitted to having poor sleep quality while 56% of students had poor sleep quality.

Analyzing the risk factors, the most of the variables analyzed in this study did not present a statistically significant relationship with poor sleep quality. Regarding gender, some studies [14, 20, 35] showed that female students present worse sleep than men; however, Li et al. [10] performed a meta-analysis involved Chinese university students and also did not verified significant difference so sleep quality between males and females, as well as others studies [3, 12, 32, 33].

Internet addiction was not associated with poor sleep quality. Different results were observed in Cheng et al. [20] study that evaluated 4318 university students in Taiwan and that verified that poor sleep quality was significantly associated with a higher tendency toward internet addition. The same measure instrument was used to evaluated the sleep quality (PSQI), but a different instrument was used to evaluated the internet addiction (Chinese Internet Addiction Scale-Revision), which may explain the differences found between this study and the Cheng et al. [20] study.

Only the presence of anxiety and/or depression were related to the quality of sleep in our study. Al-Khani et al. [4] also verified that sleep quality among medical students was significantly associated with depression (p=0. 03) and anxiety (p=0.007), as well as Montagni et al. [36] evaluated 3483 students in France and verified that the anxiety was the strongest predictor of poor sleep quality and depressive symptoms of excessive daytime sleepiness. Seun-Fadipe and Mosaku [12] used the same instrument of this study to evaluated anxiety and depression and the data revealed that the presence of symptoms of depression and anxiety were significantly associated with poor sleep quality.

This data only showed an association between the influential factors discussed above and sleep quality but did not clarify the mechanism of these influential factors, and thus the causal relationships cannot be confirmed. In the case of anxiety and depression, individuals who have these symptoms may present changes in the sleep pattern and consequently, affect its quality. That is, there was a relationship, but not the direction of this relationship, as poor sleep can lead to symptoms of anxiety and depression and vice versa.

This study presents some limitation, including the measurement instruments that despite being validated and have a high sensitivity and specificity, collect subjective information, and rely on the respondent’s self-assessment. The future studies could include objective measures, such as actigraphy, and to compare several periods of the semester when the study was conducted to verified if there are some variations in the study findings.

## Conclusions

The most of the analyzed students present a poor quality of sleep and that was associated with a presence of anxiety and depression.

It becomes necessary to develop of sleep hygiene education programs for prevention and treatment of sleep disturbances in this population, to improve students’ knowledge on the importance of adopting healthy sleep hygiene practices to enhance physical and mental health and consequently the academic performance.
